# A damage-associated molecular patterns-related gene signature for the prediction of prognosis and immune microenvironment in children stage III acute lymphoblastic leukemia

**DOI:** 10.3389/fped.2022.999684

**Published:** 2022-10-20

**Authors:** Feng Zhao, Qiuyu Lin, Xiayu Xiang, Wei Xiang

**Affiliations:** ^1^Hengyang Medical College, University of South China, Hengyang, China; ^2^Department of Pediatrics, Hengyang Maternal and Child Health Hospital, Hengyang, China; ^3^Department of Pediatrics, Hainan Women and Children’s Medical Center, Haikou, China; ^4^Peng Cheng Laboratory, Shenzhen, Guangdong, China; ^5^Commission Key Laboratory of Tropical Disease Control, Haikou, China

**Keywords:** damage-associated molecular patterns (DAMPs), immunogenic cell death, immune microenvironment, prognosis, acute lymphoblastic leukemia

## Abstract

**Background:**

Immunogenic cell death (ICD)-mediated immune response provides a strong rationale to overcome immune evasion in acute lymphoblastic leukemia (ALL). ICD will produce damage-associated molecular patterns (DAMPs) in tumor microenvironment. However, there are few studies on the application of DAMPs-related molecular subtypes in clinically predicting stage III of ALL prognosis. The current study is to identify the DAMPs-associated genes and their molecular subtypes in the stage III of ALL and construct a reliable risk model for prognosis as well as exploring the potential immune-related mechanism.

**Materials and methods:**

We used Target and EBI database for differentially expressed genes (DEGs) analysis of the stage III pediatric ALL samples. Three clusters were identified based on a consistent clustering analysis. By using Cox regression and LASSO analysis, we determined DEGs that attribute to survival benefit. In addition, the Gene Set Enrichment Analysis (GSEA) was performed to identify potential molecular pathways regulated by the DAMPs-related gene signatures. ESTIMATE was employed for evaluating the composition of immune cell populations.

**Results:**

A sum of 146 DAMPs-associated DEGs in ALL were determined and seven transcripts among them were selected to establish a risk model. The DAMPs-associated gene signature significantly contributed to worse prognosis in the high-risk group. We also found that the high-risk group exhibited low immune cell infiltration and high expression of immune checkpoints.

**Conclusion:**

In summary, our study showed that the DAMPs-related DEGs in the stage III of children ALL could be used to predict their prognosis. The risk model of DAMPs we established may be more sensitive to immunotherapy prediction.

## Introduction

Acute lymphoblastic leukemia (ALL) is the most common type of cancer that affecting children and teenagers (1, 2). The rising incidence rates of ALL are mainly caused by inherent mutations in genes that lead to uncontrolled cell growth, infectious, and environmental factors especially ionizing radiation (3–5). For childhood ALL, the peak incidence occurs at approximately 2–5 years of age and remains relatively constant before 20 years of age (6, 7). Obvious improvements in survival for ALL have been achieved in the past decades. However, progress is still slow due to the failure in the identification of genetic and/or molecular loss.

Cancer immunotherapy is an emerging therapy that activates host immune systems to eliminate tumor cells (8, 9). Recent studies have demonstrated significant clinical advances in inhibiting immune checkpoint pathways and provided a promising strategy for tumor-specific T cell response in solid tumors (10–12). Unfortunately, immune checkpoint blockade therapy has not been authorized to treat leukemia due to most patients failing to revive antitumor immune response (13–15). Based on clinical evidence of chemotherapy, the apoptosis of tumor cells can release tumor-associated antigens and sequentially stimulates an antigen-specific immune response (16, 17). Thus, tumor cell death induces the non-immunogenic tumor microenvironment transforming to immunogenic condition to mediate antitumor immunity, which is called immunogenic cell death (ICD) (18, 19). When ICD is developed in tumor microenvironment, it will produce a series of signaling molecules especially damage-associated molecular patterns (DAMPs) (20), mainly including calreticulin expressed on the cell surface, high mobility group box 1 (HMGB1) and ATP molecules secreted by cells, and heat shock proteins (HSP70, HSP90) (21–23). DAMPs released during ICD can bind to pattern recognition receptors (PRRs) on the surface of dendritic cells to initiate the downstream cell singling responses, and finally activate innate and adaptive immune responses (24–26). Reviving the patients’ own immune system to Target leukemia cells is a highly attractive treatment modality. ICD-mediated immune response provides a strong rationale to overcome immune evasion in ALL for desired therapeutic efficacy.

To understand the potency and mechanism of ICD, we collected ALL patient (0–18 years old) samples at stage III through Target and EBI database to decipher the singling molecules that govern the crosstalk between DCs and tumor cells through DAMPs. To analyze the function of DAMPs and their immune activation induced by ICD, the DAMPs-associated genes and molecular subtypes were analyzed through cell signaling pathways and immune-related responses.

## Materials and methods

### Datasets for acute lymphoblastic leukemia patients

The RNA-seq data of ALL patients at stage III (Target-ALL-P3), within 0–18 years, was downloaded from the Target dataset (27), and samples lacking survival time and survival status were removed, and finally 105 patient samples with ALL and 19611 encoding genes were obtained. The expression data of E-MTAB-1205 was obtained from the EBI database (28), and samples lacking survival time and survival status were removed, and finally 50 ALL patient samples and 21,656 genes were obtained.

### Acquisition of damage-associated molecular patterns-related genes

The DAMPs-related genes and expression pattern of protein were obtained from previous study (29) and 32 related genes were summarized in Supplementary Table 1.

### Preprocess RNA-seq data

For the Target’s RNA-seq data, we first removed the samples without clinical follow-up information such as survival time or status. Then, ensemble was converted to gene symbol and the average of the expressions with multiple gene symbols were performed. After that, we took the base 2 logarithm of the expression file (FPKM). For E-MTAB-1205 data analysis, we re-annotated the dataset through hgu133a.db in the R language to remove probes that matched one probe to multiple genes. When a gene symbol was matched with multiple probes, the mean was taken as the gene expression value.

### Damage-associated molecular patterns-associated genes for the consistent clustering of molecular subtypes in acute lymphoblastic leukemia

We used the ConsensusClusterPlus R package to determine subtypes of DAMPs-related genes *via* consistent clustering (30). Samples are classified into clusters by using the Canberra distance metric and the Pam algorithm with setting from 2 to 10. The optimal classification was determined by calculating the consistency matrix and the consistency cumulative distribution function (CDF) to obtain the molecular subtypes of the samples. Each bootstrap contained around 80% of the samples, compiling the results for 500 bootstraps. The results are shown in the heatmaps of the consistency matrix generated by the heatmap package in the R software (30, 31). Generally, the heatmaps of differential gene expression were generated by ComplexHeatmap package, in which the clusterProfiler package and anno GO_keywords were utilized to perform gene ontology (GO) enrichment analysis on the differential genes and word clouds were added to represent GO enrichment results.

### Establishment and validation of a damage-associated molecular patterns risk model

To determine the prognostic value for DAMPs-related genes, we performed the Cox regression analysis with a *P* < 0.05 and log(Fold Change) > 1.5, which were considered statistically significant. The Akaike Information Criterion (AIC) was applied for the regression analysis, which considered the statistical fit of the model and the number of parameters used for fitting by stepAIC in the MASS package (32). Generally, the method starts with the most complex model and deletes one variable in turn to reduce the AIC. The smaller AIC value indicates better efficacy of the model that obtains sufficient fit with fewer parameters. The LASSO method obtains a more refined model by constructing a penalty function and further compressing and setting some coefficients to zero (33). The advantage of subset shrinkage is retained, and it is a biased estimation for analyzing data with complex collinearity. Then, we conducted the LASSO Cox regression to reduce the gene screening scope and the prognostic significant genes were obtained (34). Lastly, DAMPs-associated genes and prognostic gene signature were determined by the multivariate Cox regression analysis. We then calculated the risk score for each patient sample using the following formula: RiskScore = Σβi × Expi, in which Expi refers to the gene expression level of the DAMPs-related phenotype and prognosis-related gene signature, and βi is the regression coefficient for the corresponding gene. Based on the threshold “0”, the patients were divided into high and low risk groups, the survival curve was drawn by the Kaplan–Meier method for prognostic analysis, and the significance was determined by using the log-rank test.

### Gene set enrichment analysis

Gene set enrichment analysis (GSEA) was performed to identify signaling pathways regulated by the different molecular subtypes (35). The Molecular Signatures Database (MSigDB) (36) was developed and utilized for GSEA analysis. All the DAMPs-related gene candidates from KEGG pathway were analyzed by GSEA through clusterProfiler package (37). *P-*value (calculated by R software) smaller than 0.05 was determined as statistically significant. The correlation coefficients were also calculated by R.

### Evaluation of immune cell abundance in tumor microenvironment

We used the ssGSEA algorithm (38) to quantify the relative abundance of immune cells in tumor tissue. Meanwhile, we also utilized the ESTIMATE (39) to calculate the fractions of immune cell types between low- and high-risk groups. The immune cell type score of each sample was estimated by MCPcounter package (40).

### Prediction of responsiveness to immunotherapy

We used the tumor immune dysfunction and exclusion (TIDE) algorithm^1^ to verify the effect of immune checkpoint inhibitor score (IMS) in the prediction of clinical responsiveness to immune checkpoint blockade (ICB). The TIDE algorithm is a computational method for predicting ICB responsiveness using gene expression profiling (41).

### Differential expression analysis

Limma package was used to identified differentially expressed genes (DEGs) with threshold value logFC > 1.5 and *p* < 0.05.

### Statistical analysis

The data analysis in this study was supported by Sangerbox platform (42). Data were expressed as the mean ± standard deviation (s.d.). Statistical significance was determined by Wilcox test through R software when different groups were compared. The comparison between different DAMP subgroups were performed by the Kruskal-Wallis test. Kaplan Meier curves were plotted to show the survival time differences. Log-rank Mantel-Cox test was used for survival curves.

## Results and discussion

### Using the damage-associated molecular patterns-associated genes for the consistent clustering of molecular subtypes in acute lymphoblastic leukemia

**FIGURE 1:**
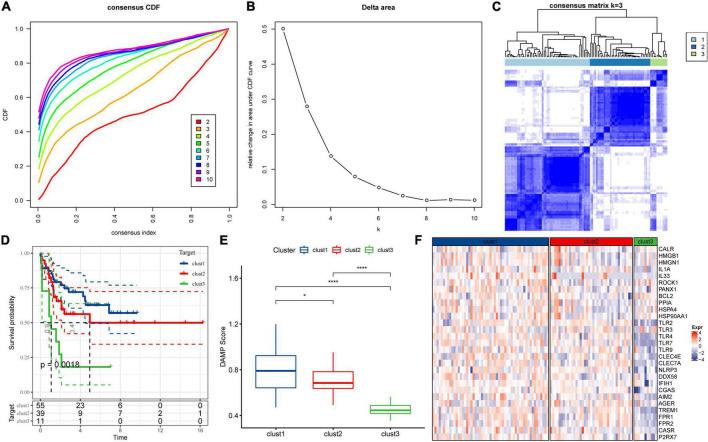
Consensus clustering of molecular subtypes in acute lymphoblastic leukemia depending on damage-associated molecular patterns-associated differentially expressed genes. **(A)** Cumulative distribution function (CDF) curve of Target dataset samples. **(B)** CDF Delta area curve. The relative change in the area under the curve for each category number k in comparison with k−1 is displayed. The number *k* is indicated by the horizontal axis and the relative change in the area under the curve is represented by the vertical axis. **(C)** The heatmap for the consensus matrix with *k* = 3. **(D)** K-M survival curves showed the differences of among the three clusters. **(E)** Comparison of DAMPs scores of the three subtypes in the Target dataset. **(F)** Heatmap of DAMPs-related gene expression between different molecular subtypes. *p < 0.05; **p < 0.01; ***p < 0.001; ****p < 0.0001; ns: no significance.

To understand the expression patterns of DAMPs-related genes in ALL, the tumor tissue samples were obtained from the Target dataset containing clinical information and 32 DAMPs-related genes. Among these genes, 29 genes were detected in the Target dataset, while P2Y2R, P2Y6R, and P2Y12R were not identified. Then we constructed consistent clustering of 29 DAMPs-related gene expression profiles. CDF was utilized to decide the optimal cluster number. When *k* = 3, the clustering output was relatively consistent (Figure 1A). The CDF delta area curve analysis showed similar trend after three clusters (Figure 1B). We therefore selected *k* = 3 to obtain three molecular subtypes for further analysis. The heatmap of DAMPs-related genes in the three clusters was shown in Figure 1C. Further prognostic analysis of these three subtypes were performed. The results showed the significant differences in survival probability among these three subtypes. Compared to the other two clusters, the samples in clust1 had the best prognosis (Figure 1D). We further calculated DAMPs-related scores by using ssGSEA method. As shown in Figure 1E, we found that the clust1 subtype with the best prognosis had a significantly higher DAMPs score than the other two subtypes. The heatmap of DAMPs-related gene expression in these three isoforms was shown in Figure 1F, and most genes were relatively low expression in the clust3 subtype.

### Pathway analysis for molecular subtypes in acute lymphoblastic leukemia

**FIGURE 2:**
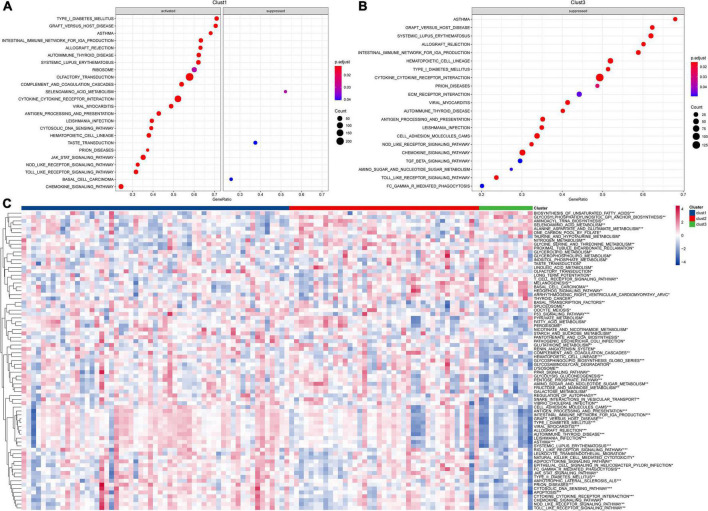
Pathway analysis for molecular subtypes in ALL. **(A)** GSEA analysis of clust1 vs. no_clust1 in the Target dataset; **(B)** GSEA analysis of clust3 vs. no_clust3 in the Target dataset; **(C)** GSVA analysis of pathway enrichment scores for each of the three subtypes (*P < 0.05; **P < 0.01; ***P < 0.001; and ****P < 0.0001).

To better understand the functional characteristics of these three clusters, we performed differential expression analysis through the limma package and sorted the genes based on log2 fold change (LogFC > 1.5, *P* < 0.05) values. GSEA was further performed based on differentially expressed genes (DEGs) for each cluster to analyze KEGG pathway. The results showed that cluster-2 has no significantly regulated pathways, and clust1 has three inhibited pathways and 21 activated pathways, while 21 inhibited pathways were shown in clust3 (Figures 2A,B). Particularly, compared to clust3, clust1 showed significant activation of immune-related signaling pathways including cytokine and its receptor interaction, Toll like receptor downstream signaling, chemokine signaling pathways and intestinal immune network for IGA production, indicating the immune response to ALL tumor in clust1. Next, we calculated each patient’s score for each KEGG pathway using the ssGSEA method under the GSVA package. Each pathway’s scores across three clusters were compared. Pathways with *p*-values smaller than 0.05 are selected as key pathways. As shown in Figure 2C, clust3 showed suppressed immune-related signaling pathways due its poor prognosis, while clust1 has obvious activation of immune associated pathways in ALL tumor microenvironment.

### Immune signature of molecular subtypes in acute lymphoblastic leukemia

**FIGURE 3:**
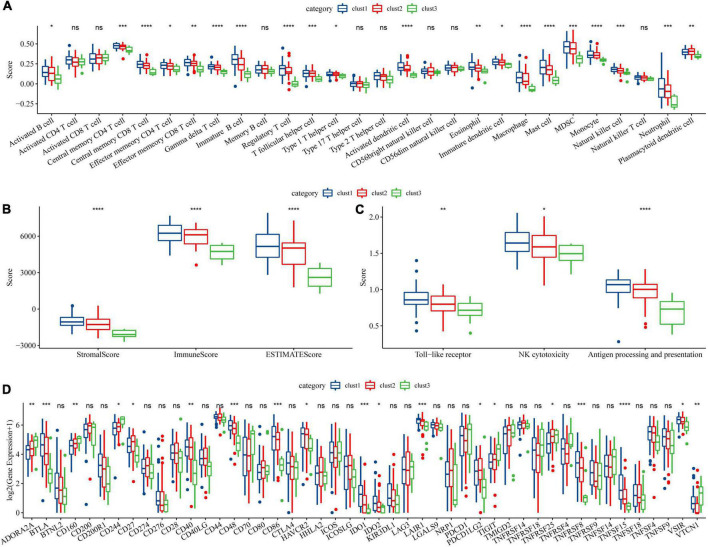
Immune signature among molecular subtypes of ALL in the Target dataset. **(A)** Differences of 28 types of immune cell scores between different molecular subtypes; **(B)** Differences in immune infiltration between different molecular subtypes through ESTIMATE; **(C)** Differential analysis of macrophage-involved pathways between different subtypes; **(D)** Differentially expressed immune checkpoints between different subtypes (*P < 0.05; **P < 0.01; ***P < 0.001; and ****P < 0.0001).

To further elucidate the differences in the immune microenvironment of patients between different molecular subtypes, we first calculated the scores of 28 immune cells by the ssGSEA method through immune cell-related gene sets (38). We found significant differences in some immune cells such as macrophages among these three subtypes (Figure 3A). Moreover, the immune score in each sample was calculated by the ESTIMATE method and the increased immune infiltration inside tumor tissues was observed in the clust1 subtype with the best prognosis (Figure 3B). Tumor-associated macrophages play a critical role in immune regulation including the capability of antigen presentation, Toll-like receptor signaling pathway-induced immune activation and FC receptors existing on the surface of macrophages, which can kill tumor cells through natural killer (NK) cell-mediated specific antibody-dependent cellular cytotoxicity (ADCC). As shown in Figure 3A, we found significant change in the macrophages among the three molecular subtypes. Therefore, we used the ssGSEA method to calculate the score of Toll like receptor core, NK cytotoxicity, and antigen processing and presentation of each sample through the signaling pathways. As shown in Figure 3C, there were significant differences in the macrophage-related scores of the three subtypes. Finally, we downloaded the immune checkpoint-related genes reported from previous study (43) and compared the expression of immune checkpoints in the three subtypes (Figure 3D). We found significant differences in the expression of some immune checkpoints among the three subtypes. Our findings indicated that immune microenvironment and their related signaling pathways may be responsible for the prognosis of ALL among the three DAMPs-related molecular subtypes.

### Identification of key genes for damage-associated molecular patterns phenotype and construction of damage-associated molecular patterns-related risk models

In the previous analysis, we have identified three molecular subtypes based on DAMPs-associated genes and found differences among the subtypes in immune signatures and pathways. Among the three subtypes, clust3 had a poor prognosis, clust2 was the second, and clust1 had the best prognosis. Then, we performed differential expression analysis on clust1 vs. no_clust1 subtypes, clust2 vs. no_clust2, clust3 vs. no_clust3 subtypes to screen differential gens. In clust1 vs. no_clust1, 379 genes were upregulated, and 90 downregulated genes were screened, while 109 upregulated genes and 86 downregulated genes were found in clust2 vs. no_clust2. There were 821 upregulated genes and 1893 downregulated genes in clust3 vs. no_clust3. The volcano plots of difference analysis were shown in Supplementary Figures 1A,C,E. Finally, we screened a total of 2,927 differential genes for further analysis. The heatmaps of differential gene expression were generated (Supplementary Figures 1B,D,F).

**FIGURE 4:**
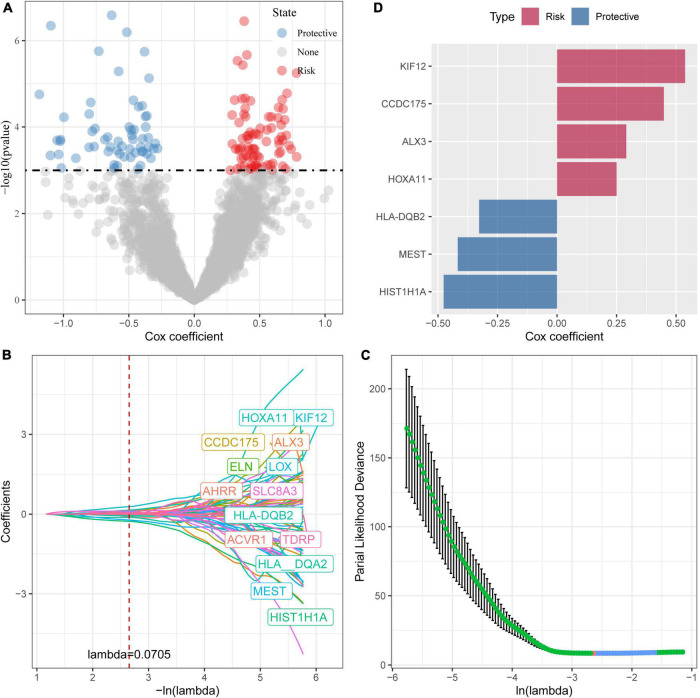
Identification of prognostic genes. **(A)** A total of 961 promising candidates were identified among the DEGs; **(B)** The trajectory-based change of each independent variable with lambda; **(C)** The optimal λ selection by cross-validated deviance of LASSO fit; **(D)** Multivariate cox analysis and the coefficients of prognosis-related genes.

Next, we performed univariate cox analysis on the 2,927 differential genes and identified a total of 146 genes with a significant impact on prognosis (*P* < 0.001), including 85 genes in Risk group and 61 genes in Protective group (Figure 4A). We further performed a LASSO-Cox regression analysis to shrink the scope of gene screening among these 146 key genes. As shown in Figure 4B, the trajectory-based change of each independent variable was analyzed. With the gradual increase of lambda, the number of independent variable coefficients tending to zero also gradually increased. The penalty parameter was established through 10-fold cross validation and the confidence interval under each lambda was analyzed. As shown in Figure 4C, the model reaches the optimum when lambda is 0.0706. Therefore, we selected 14 genes as the Target genes and further performed stepwise multivariate regression analysis. As shown in Figure 4D, by analyzing a multivariate Cox regression, seven genes (HLA-DQB2, HIST1H1A, MEST, ALX3, KIF12, CCDC175, and HOXA11) were selected to build a gene signature model as follows:


$$\begin{array}{l} RiskScore=-0.327\times HLA-DQB2-0.476\times HIST1H1A \end{array}$$

-0.417×M⁢E⁢S⁢T+0.291×A⁢L⁢X⁢3+0.538

×K⁢I⁢F⁢12+0.449×C⁢C⁢D⁢C⁢175+0.25

×H⁢O⁢X⁢A⁢11.

**FIGURE 5:**
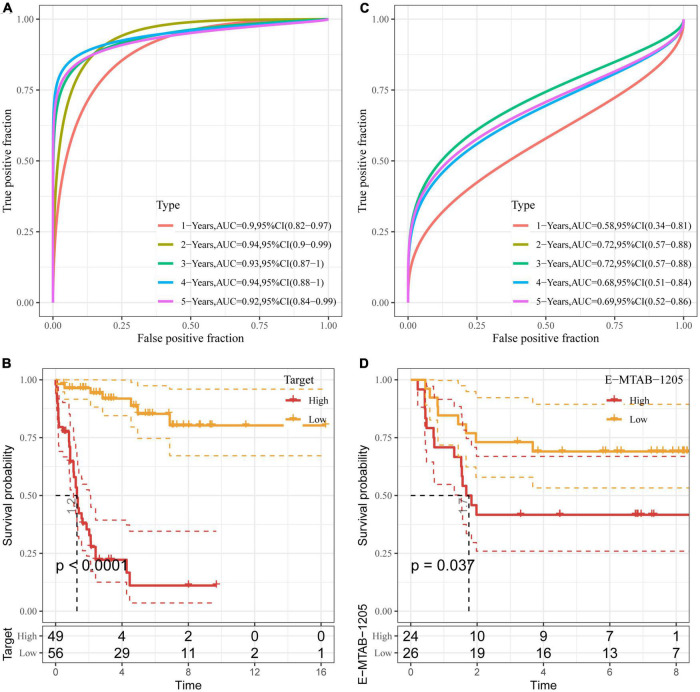
Validation of DAMPs-related risk model. **(A,B)** ROC curve **(A)** and K**–**M survival curve **(B)** of risk model constructed by seven genes in Target dataset; **(C,D)** ROC curve **(C)** and K–M survival **(D)** of risk model constructed by seven genes in E-MTAB-1205 dataset.

We then used the Target data as the training data set and the risk score of each sample was calculated based on the expression levels of the seven genes. As shown in Figure 5A, the classification efficiency of prognostic prediction demonstrated that the area under the time-dependent ROC curves (AUC) reached 0.9 in 1–5 years, indicating the predictive capability of this model. *Z*-score was also performed on RiskScore and the samples with RiskScore more than zero were assigned into high-risk group, and the samples with less than zero risk belonged to low-risk group. As shown in Figure 5B, the low-risk group showed excellent survival benefit compared to high-risk group (*p* < 0.0001).

To better verify the robustness of the model, we used the E-MTAB-1205 dataset for validation and the risk model established by these seven genes was used to perform prognostic classification on RiskScore. As shown in Figure 5C, the classification efficiency of prognosis prediction was analyzed in 1–5 years, and the AUC reached 0.7 in 2 and 3 years. As shown in Figure 5D, the K–M curve of low-risk group showed prolonged survival time than that of high-risk group (*P* < 0.05).

### Clinical phenotypic differences between damage-associated molecular patterns-related subtypes and risk models

Then, we showed the distribution of high- and low-risk groups in age, gender, and DAMPs type by Sankey diagram (Supplementary Figure 2A). The difference in RiskScore between age, gender, and DAMPs type was further analyzed. The results showed that clust3 with the worst prognosis had higher risk values compared to the other two subtypes. Moreover, patients with death status showed higher risk scores than those in alive group (Supplementary Figure 2B), indicating the risk model we established showed high sensitivity and specificity.

### RiskScore analysis of immune microenvironment and signaling pathways

**FIGURE 6:**
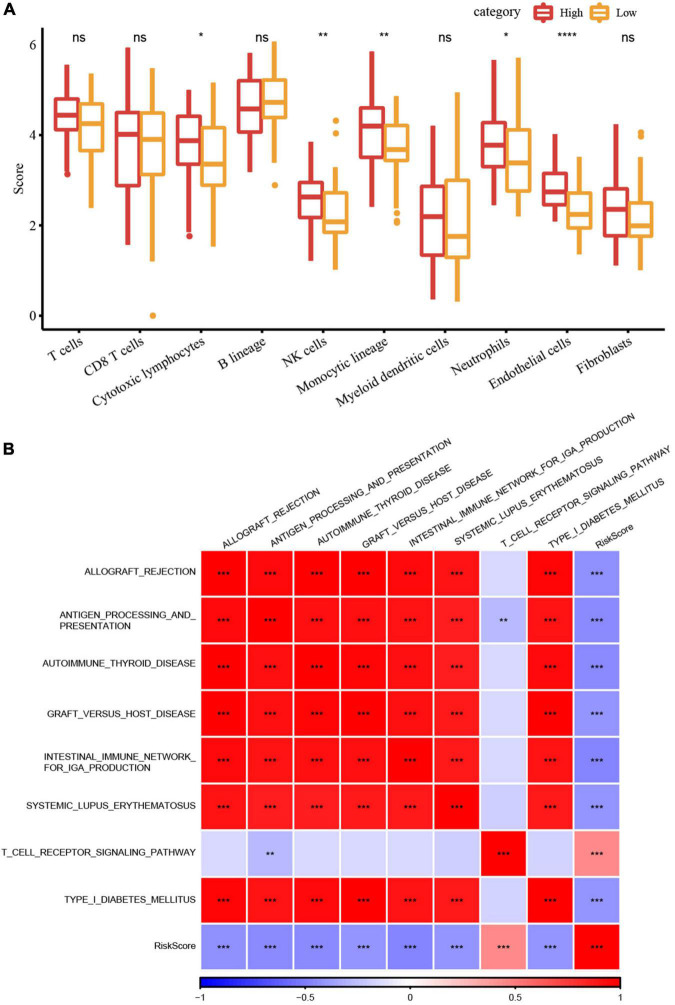
RiskScore analysis of immune microenvironment and signaling pathways. **(A)** Differences in 10 different cell types predicted by the MCPcounter method between high- and low-risk groups; **(B)** Heatmap of the correlation analysis between risk scores and potential immune modulations (*P < 0.05; **P < 0.01; ***P < 0.001; and ****P < 0.0001).

We first analyzed the immune checkpoint-related gene expressions between high- and low-risk groups in the Target dataset and found that some immune checkpoints showed significant differences between these two groups. The correlation analysis between 28 immune cell scores and risk scores were performed and we found that some immune cells including activated B cells, NK cells, effector memory CD4 and CD8 T cells were negatively correlated with risk scores (Supplementary Figure 3), indicating that high immune cell infiltration may suppress the tumor progression. Further, we calculated the scores of 10 different cell types through MCP-counter. The scores of some cell types such as NK cells, monocytic lineage, neutrophils, and endothelial cells were significantly different between high- and low-risk groups (Figure 6A).

Finally, we used the GSVA package (44) to estimate the pathway score of each sample for each KEGG pathway. The correlation analysis between these pathways and their risk score through Hmisc package (cor > 0.4 and *p* < 0.001) was performed to screen the pathways significantly related to the risk score. Figure 6B showed eight signaling pathways were significantly correlated with the risk score, of which the T cell receptor signaling pathway was positively correlated, while the other seven pathways had significant negative correlations.

### Correlation analysis between the expression of key damage-associated molecular patterns-related genes in risk models and immune infiltration

**FIGURE 7:**
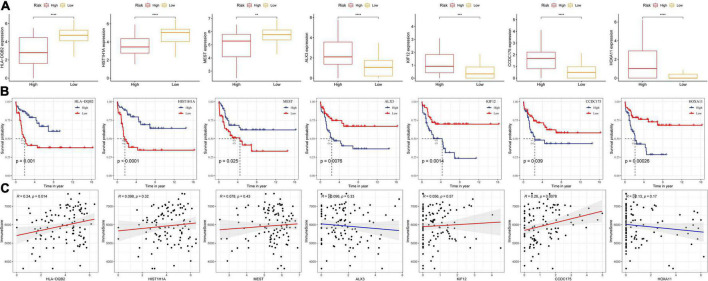
Correlation analysis between the expression of key DAMPs-related genes in risk models and immune infiltration. **(A)** The expression of seven key genes in high- and low- risk groups; **(B)** K–M survival curve of high- and low- gene expression group; **(C)** Pearson correlation analysis between gene expression and immune score (*P < 0.05; **P < 0.01; ***P < 0.001; and ****P < 0.0001).

We established a risk model for seven key DAMPs-related genes and found no significant difference in the distribution of risk models in terms of age and gender, which indicated that these two factors were not associated with ICD in ALL disease. To better analyze the DAMPs-related risk model, data showed that four of the seven genes belong to risk group and the other three genes were related to protective group (Figure 7A). Moreover, three protective-related genes were highly expressed in the low-risk group, while the expression of other risk-related genes were increased in the high-risk group. We further analyzed their survival benefit and the results showed that three protective-related genes with low expression or four risk-related genes with high expression had a worse prognosis than that in other groups (Figure 7B). We further calculated the immune score of patients by ESTIMATE and the relationship between these seven genes and immune infiltration were estimated by Pearson’s correlation analysis (Figure 7C), in which HLA-DQB2 and CCDC175 genes were significantly positively correlated with immune infiltration.

### Performance comparison between risk model and tumor immune dysfunction and exclusion

**FIGURE 8:**
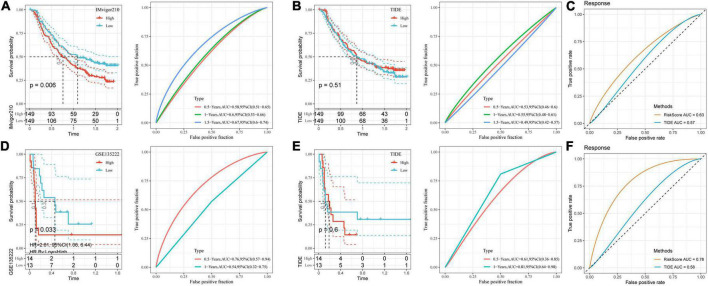
Performance comparison between risk model and TIDE. **(A)** K–M curve and ROC curve of DAMPs risk score in IMvigor210 dataset; **(B)** K–M curve and ROC curve of immunotherapy response predicted by TIDE in IMvigor210 dataset; **(C)** DAMPs risk score in IMvigor210 dataset and ROC curve of immunotherapy effect predicted by TIDE; **(D)** K–M curve and ROC curve of DAMPs risk score in GSE135222 dataset; **(E)** K–M curve and ROC curve of immunotherapy response predicted by TIDE in GSE135222 dataset; **(F)** DAMPs risk score of GSE135222 dataset and ROC curves of immunotherapy effect predicted by TIDE.

Through the existing analysis, we collected the clinical samples after immunotherapy (IMvigor210 and GSE135222) and constructed DAMPs risk model scores by using these seven screened genes. The online tool TIDE was utilized to evaluate the TIDE score of immunotherapy effect. As shown in Figures 8A,D, we divided the high and low risk groups by the median value using the model we established and found poor prognosis in high-risk group. Next, we compared the prognosis of the response to immunotherapy predicted by TIDE between the two datasets. The result showed no significant difference in the prognosis (Figures 8B,E). We further calculated the AUC of the DAMPs risk model and TIDE on the effect of immunotherapy and found that the effect of the DAMPs risk model on immunotherapy was better than that of TIDE (Figures 8C,F).

## Discussion

Acute lymphoblastic leukemia is one of the leading causes of cancer-related mortality in children and teenagers. Extensive studies have demonstrated that various factors such as genetic condition and radiation/chemical exposures contribute to ALL development (45). While improved survival for adult ALL have been achieved during the past decades, the prognosis for pediatric ALL is still poor due to limited understanding in disease-related gene signatures and/or failure in determination of certain molecular loss. Bioinformatic analysis on next-generation sequencing data has recently emerged as a powerful tool for the exploration of molecular mechanisms, which allows to determine various molecular subtypes and gene signatures as well as facilitates the establishment of risk models for disease development prediction. By using RNAseq data generated from pediatric (0–18 years old) ALL patient samples, we performed gene signature analysis, identified DAMPs-related subtypes and signaling pathways, and established risk prediction models and scoring systems.

Herein, we found that clust1 was associated with better prognosis and responsiveness to immunotherapy. A sum of 146 DAMPs-associated DEGs in pediatric ALL samples were identified. By screening of gene signatures, we obtained seven significant genes including HLA-DQB2, HIST1H1A, MEST, ALX3, KIF12, CCDC175, and HOXA11, showing robust correlation with ALL development. Among these genes, the partial control of human leucocyte antigen (HLA) genes like HLA-DQB2 on immune response to infection (45, 46) could be used as a reasonable therapeutic target, which will be further explored to revive the antitumor immunity in ALL. Some studies have demonstrated that MEST promotes invasion and metastasis of solid tumors and by coordinating and activating the NF-κB and Wnt/β-catenin signaling pathways (47, 48), which is consistent with our findings of MEST gene and its related signaling pathways in ALL. The functional and clinical significance of MEST and its potential target against ALL could be considered. Overall, the correlation between above mentioned genes and ALL prognosis may provide potential molecular targets for the treatment of pediatric ALL patients.

We further established a prognostic model based on those genes and performed validation studies to test the prediction efficiency on high- and low-risk groups. The results showed that DAMPs-associated gene signature was significantly associated with worse prognosis in the high-risk group. We also found that the high-risk group exhibited lower immune cell infiltration and higher expression of immune checkpoints compared to low-risk group. The tumor-infiltrating immune cells and immune scores were estimated by ESTIMATE and ssGSEA for each ALL-patient sample. We found that the activated B cells, NK cells, effector memory CD4 and CD8 T cells was enriched in the low-risk group and correlated with good clinical outcome. Patients with higher effector T cells, NK cells, and B cells infiltration showed better prognosis, which was similar with previous observations (49, 50). As is reported, high expression of immune checkpoints indicates an immunosuppressive microenvironment in the tumor (51). High-risk patients showed up-regulation of immune checkpoints-related signaling pathways, which is associated with poor survival benefit and low immune cell infiltration inside ALL. Collectively, the model we established has also been evaluated by clinical datasets of immunotherapy which showed robust antitumor immunity in the low-risk group than that in the high-risk group. The results demonstrated that our risk model had high accuracy and sensitivity to predict the prognosis.

The findings here await further validation in experimental settings, which can be a potential future research direction. Additionally, the comprehensive tumor microenvironment within pediatric ALL including the interaction between tumor cells and immune cells is another promising direction worth further exploration.

## Conclusion

In this study, three molecular subtypes related to prognosis were constructed by DAMPs-related genes and their function and immune-related pathways in stage III ALL were analyzed through signaling pathway and immune analysis. The seven key DAMPs-related genes were analyzed and screened through univariate cox analysis, LASSO, and stepwise regression. Finally, a risk model was constructed through multivariate cox analysis. The clinical phenotype differences, immune and pathway characteristics were used to estimate the DAMPs risk model, which was further verified by two clinical datasets in immunotherapy. The risk model of DAMPs we established may be more sensitive to immunotherapy prediction.

## Data availability statement

The datasets presented in this study can be found in online repositories. The names of the repository/repositories and accession number(s) can be found in the article/Supplementary material.

## Author contributions

FZ designed the study and acquired the data. QL drafted the manuscript. WX and XX revised the manuscript. All authors contributed to this work and read and approved the manuscript.

## Footnotes

1
http://tide.dfci.harvard.edu/

## References

[1] Hunger SP Mullighan CG. Acute lymphoblastic leukemia in children. *N Engl J Med.* (2015) 373:1541–52. 10.1056/NEJMra1400972 26465987

[2] Seibel NL. Treatment of acute lymphoblastic leukemia in children and adolescents: peaks and pitfalls. *Am Soc Hematol Educ Program Book.* (2008) 2008:374–80. 10.1182/asheducation-2008.1.374 19074113

[3] Redaelli A Laskin BL Stephens JM Botteman MF Pashos CL. A systematic literature review of the clinical and epidemiological burden of acute lymphoblastic leukaemia (ALL). *Eur J Cancer Care.* (2005) 14:53–62. 10.1111/j.1365-2354.2005.00513.x 15698386

[4] Malard F Mohty M. Acute lymphoblastic leukaemia. *Lancet.* (2020) 395:1146–62. 10.1016/S0140-6736(19)33018-1 32247396

[5] Richardson DB Wing S Schroeder J Schmitz-Feuerhake I Hoffmann W. Ionizing radiation and chronic lymphocytic leukemia. *Environ Health Perspect.* (2005) 113:1–5. 10.1289/ehp.7433 15626639 PMC1253701

[6] Bassan R Gatta G Tondini C Willemze R. Adult acute lymphoblastic leukaemia. *Crit Rev Oncol Hematol.* (2004) 50:223–61. 10.1016/j.critrevonc.2003.11.003 15182827

[7] Wiemels J Cazzaniga G Daniotti M Eden OB Addison GM Masera G Prenatal origin of acute lymphoblastic leukaemia in children. *Lancet.* (1999) 354:1499–503. 10.1016/S0140-6736(99)09403-9 10551495

[8] Whiteside TL Demaria S Rodriguez-Ruiz ME Zarour HM Melero I. Emerging opportunities and challenges in cancer immunotherapy. *Clin Cancer Res.* (2016) 22:1845–55. 10.1158/1078-0432.CCR-16-0049 27084738 PMC4943317

[9] Yuan J Hegde PS Clynes R Foukas PG Harari A Kleen TO Novel technologies and emerging biomarkers for personalized cancer immunotherapy. *J Immunother Cancer.* (2016) 4:3. 10.1186/s40425-016-0107-3 26788324 PMC4717548

[10] Akinleye A Rasool Z. Immune checkpoint inhibitors of PD-L1 as cancer therapeutics. *J Hematol Oncol.* (2019) 12:92. 10.1186/s13045-019-0779-5 31488176 PMC6729004

[11] Sharma P Allison JP. The future of immune checkpoint therapy. *Science.* (2015) 348:56–61. 10.1126/science.aaa8172 25838373

[12] Arora S Velichinskii R Lesh RW Ali U Kubiak M Bansal P Existing and emerging biomarkers for immune checkpoint immunotherapy in solid tumors. *Adv Ther.* (2019) 36:2638–78. 10.1007/s12325-019-01051-z 31410780 PMC6778545

[13] Daver N Boddu P Garcia-Manero G Yadav SS Sharma P Allison J Hypomethylating agents in combination with immune checkpoint inhibitors in acute myeloid leukemia and myelodysplastic syndromes. *Leukemia.* (2018) 32:1094–105. 10.1038/s41375-018-0070-8 29487386 PMC6916728

[14] Jimbu L Mesaros O Popescu C Neaga A Berceanu I Dima D Is there a place for PD-1-PD-L blockade in acute myeloid leukemia? *Pharmaceuticals.* (2021) 14:288. 10.3390/ph14040288 33804850 PMC8063836

[15] Wang H Kaur G Sankin AI Chen F Guan F Zang X Immune checkpoint blockade and CAR-T cell therapy in hematologic malignancies. *J Hematol Oncol.* (2019) 12:59. 10.1186/s13045-019-0746-1 31186046 PMC6558778

[16] Nesslinger NJ Sahota RA Stone B Johnson K Chima N King C Standard treatments induce antigen-specific immune responses in prostate cancer. *Clin Cancer Res.* (2007) 13:1493–502. 10.1158/1078-0432.CCR-06-1772 17332294

[17] Hänel G Angerer C Petry K Lichtenegger FS Subklewe M. Blood DCs activated with R848 and poly (I: C) induce antigen-specific immune responses against viral and tumor-associated antigens. *Cancer Immunol Immunother.* (2022) 71:1705–18. 10.1007/s00262-021-03109-w 34821951 PMC8614222

[18] Kepp O Zitvogel L. Immunogenic cell death in cancer therapy. *Annu Rev Immunol.* (2013) 31:51–72. 10.1146/annurev-immunol-032712-100008 23157435

[19] Galluzzi L Buqué A Kepp O Zitvogel L Kroemer G. Immunogenic cell death in cancer and infectious disease. *Nat Rev Immunol.* (2017) 17:97–111. 10.1038/nri.2016.107 27748397

[20] Krysko DV Garg AD Kaczmarek A Krysko O Agostinis P Vandenabeele P Immunogenic cell death and DAMPs in cancer therapy. *Nat Rev Cancer.* (2012) 12:860–75. 10.1038/nrc3380 23151605

[21] Fucikova J Moserova I Truxova I Hermanova I Vancurova I Partlova S High hydrostatic pressure induces immunogenic cell death in human tumor cells. *Int J Cancer.* (2014) 135:1165–77. 10.1002/ijc.28766 24500981

[22] Ahmed A Tait SW. Targeting immunogenic cell death in cancer. *Mol Oncol.* (2020) 14:2994–3006. 33179413 10.1002/1878-0261.12851 PMC7718954

[23] Garg AD Nowis D Golab J Vandenabeele P Krysko DV Agostinis P Immunogenic cell death, DAMPs and anticancer therapeutics: an emerging amalgamation. *Biochim Biophys Acta.* (2010) 1805:53–71. 10.1016/j.bbcan.2009.08.003 19720113

[24] Amarante-Mendes GP Adjemian S Branco LM Zanetti LC Weinlich R Bortoluci KR Pattern recognition receptors and the host cell death molecular machinery. *Front Immunol.* (2018) 9:2379. 10.3389/fimmu.2018.02379 30459758 PMC6232773

[25] Fucikova J Kepp O Kasikova L Petroni G Yamazaki T Liu P Detection of immunogenic cell death and its relevance for cancer therapy. *Cell Death Dis.* (2020) 11:1013. 33243969 10.1038/s41419-020-03221-2 PMC7691519

[26] Garg AD Dudek AM Agostinis P. Cancer immunogenicity, danger signals, and DAMPs: what, when, and how? *Biofactors.* (2013) 39:355–67. 10.1002/biof.1125 23900966

[27] Zhou C Tang J Yuan Y Ren Y Xiao X Zheng L Space-time array difference magnetotelluric method. *Proceedings of the 2016 Progress in Electromagnetic Research Symposium (PIERS).* Shanghai: IEEE (2016).

[28] Qi H Chi L Wang X Jin X Wang W Lan J Identification of a seven-lncRNA-mRNA signature for recurrence and prognostic prediction in relapsed acute lymphoblastic leukemia based on WGCNA and LASSO analyses. *Analyt Cell Pathol.* (2021) 2021:6692022. 10.1155/2021/6692022 34211824 PMC8208884

[29] Xu M Lu JH Zhong YZ Jiang J Shen YZ Su JY Immunogenic cell death-relevant damage-associated molecular patterns and sensing receptors in triple-negative breast cancer molecular subtypes and implications for immunotherapy. *Front Oncol.* (2022) 12:870914. 10.3389/fonc.2022.870914 35444934 PMC9013947

[30] Wilkerson MD Hayes DN. ConsensusClusterPlus: a class discovery tool with confidence assessments and item tracking. *Bioinformatics.* (2010) 26:1572–3. 10.1093/bioinformatics/btq170 20427518 PMC2881355

[31] Gu Z Eils R Schlesner M. Complex heatmaps reveal patterns and correlations in multidimensional genomic data. *Bioinformatics.* (2016) 32:2847–9. 10.1093/bioinformatics/btw313 27207943

[32] Schweitzer S Kunz M Kurlbaum M Vey J Kendl S Deutschbein T Plasma steroid metabolome profiling for the diagnosis of adrenocortical carcinoma. *Eur J Endocrinol.* (2019) 180:117–25. 30481155 10.1530/EJE-18-0782

[33] Shahraki HR Salehi A Zare N. Survival prognostic factors of male breast cancer in Southern Iran: a LASSO-Cox regression approach. *Asian Pacif J Cancer Prev.* (2015) 16:6773–7. 10.7314/apjcp.2015.16.15.6773 26434910

[34] Zhang B Tang B Gao J Li J Kong L Qin L A hypoxia-related signature for clinically predicting diagnosis, prognosis and immune microenvironment of hepatocellular carcinoma patients. *J Transl Med.* (2020) 18:342. 10.1186/s12967-020-02492-9 32887635 PMC7487492

[35] Liberzon A Birger C Thorvaldsdóttir H Ghandi M Mesirov JP Tamayo P The molecular signatures database hallmark gene set collection. *Cell Syst.* (2015) 1:417–25. 10.1016/j.cels.2015.12.004 26771021 PMC4707969

[36] Liberzon A Subramanian A Pinchback R Thorvaldsdóttir H Tamayo P Mesirov JP Molecular signatures database (MSigDB) 3.0. *Bioinformatics.* (2011) 27:1739–40. 10.1093/bioinformatics/btr260 21546393 PMC3106198

[37] Wu T Hu E Xu S Chen M Guo P Dai Z clusterProfiler 4.0: a universal enrichment tool for interpreting omics data. *Innovation.* (2021) 2:100141. 10.1016/j.xinn.2021.100141 34557778 PMC8454663

[38] Charoentong P Finotello F Angelova M Mayer C Efremova M Rieder D Pan-cancer immunogenomic analyses reveal genotype-immunophenotype relationships and predictors of response to checkpoint blockade. *Cell Rep.* (2017) 18:248–62. 10.1016/j.celrep.2016.12.019 28052254

[39] Yoshihara K Shahmoradgoli M Martínez E Vegesna R Kim H Torres-Garcia W Inferring tumour purity and stromal and immune cell admixture from expression data. *Nat Commun.* (2013) 4:2612. 10.1038/ncomms3612 24113773 PMC3826632

[40] Becht E Giraldo NA Lacroix L Buttard B Elarouci N Petitprez F Estimating the population abundance of tissue-infiltrating immune and stromal cell populations using gene expression. *Genome Biol.* (2016) 17:218. 10.1186/s13059-016-1070-5 27765066 PMC5073889

[41] Jiang P Gu S Pan D Fu J Sahu A Hu X Signatures of T cell dysfunction and exclusion predict cancer immunotherapy response. *Nat Med.* (2018) 24:1550–8. 30127393 10.1038/s41591-018-0136-1 PMC6487502

[42] Shen W Song Z Zhong X Huang M Shen D Gao P Sangerbox: a comprehensive, interaction-friendly clinical bioinformatics analysis platform. *iMeta.* (2022) 1:e36. 10.1002/imt2.36 PMC10989974 38868713

[43] Danilova L Ho WJ Zhu Q Vithayathil T De Jesus-Acosta A Azad NS Programmed cell death ligand-1 (PD-L1) and CD8 expression profiling identify an immunologic subtype of pancreatic ductal adenocarcinomas with favorable survival. *Cancer Immunol Res.* (2019) 7:886–95. 10.1158/2326-6066.CIR-18-0822 31043417 PMC6548624

[44] Alhamdoosh M Law CW Tian L Sheridan JM Ng M Ritchie ME Easy and efficient ensemble gene set testing with EGSEA. *F1000Research.* (2017) 6:2010. 10.12688/f1000research.12544.1 29333246 PMC5747338

[45] Linet MS Schubauer-Berigan MK Weisenburger DD Richardson DB Landgren O Blair A Chronic lymphocytic leukaemia: an overview of aetiology in light of recent developments in classification and pathogenesis. *Br J Haematol.* (2007) 139:672–86. 10.1111/j.1365-2141.2007.06847.x 18021081

[46] Dearden S Taylor GM Gokhale DA Robinson MD Thompson W Ollier W Molecular analysis of HLA-DQB1 alleles in childhood common acute lymphoblastic leukaemia. *Br J Cancer.* (1996) 73:603–9. 10.1038/bjc.1996.104 8605093 PMC2074350

[47] Wang Y Zhang J Yi YJ Yu NN Liu WT Liang JZ MEST promotes lung cancer invasion and metastasis by interacting with VCP to activate NF-κB signaling. *J Exp Clin Cancer Res.* (2021) 40:301. 10.1186/s13046-021-02107-1 34560900 PMC8464132

[48] Chen L Wu X Xie H Yao N Xia Y Ma G ZFP57 suppress proliferation of breast cancer cells through down-regulation of MEST-mediated Wnt/β-catenin signalling pathway. *Cell Death Dis.* (2019) 10:169. 10.1038/s41419-019-1335-5 30787268 PMC6382817

[49] Liu X Chen J Lu W Zeng Z Li J Jiang X Systematic profiling of immune risk model to predict survival and immunotherapy response in head and neck squamous cell carcinoma. *Front Genet.* (2020) 11:576566. 10.3389/fgene.2020.576566 33193693 PMC7596453

[50] Gonzalez-Rodriguez AP Contesti J Huergo-Zapico L Lopez-Soto A Fernández-Guizán A Acebes-Huerta A Prognostic significance of CD8 and CD4 T cells in chronic lymphocytic leukemia. *Leuk Lymphoma.* (2010) 51:1829–36. 10.3109/10428194.2010.503820 20846097

[51] Hu M Wang Y Xu L An S Tang Y Zhou X Relaxin gene delivery mitigates liver metastasis and synergizes with check point therapy. *Nat Commun.* (2019) 10:2993. 10.1038/s41467-019-10893-8 31278269 PMC6611764

