# Supplementary material for: A damage-associated molecular patterns-related gene signature for the prediction of prognosis and immune microenvironment in children stage III acute lymphoblastic leukemia
Source: Front Pediatr. 2022 Oct 20;10:999684. doi: 10.3389/fped.2022.999684 (PMC9631945; doi:10.3389/fped.2022.999684)
Supplement: Supplementary file 1 [file Data_Sheet_1.docx]

**Supplementary materials**

**Fig.S1:** Identification of key genes for DAMPs phenotype and establishment of DAMPs-related risk models. (A) Volcano plot of differential analysis of clust1 vs no_clust1; (B) Heatmap of differential gene expression of clust1 vs no_clust1; (C) Volcano plot of differential analysis of clust2 vs no_clust2; (D) Heatmap of differential gene expression of clust2 vs no_clust2; (E) Volcano plot of clust3 vs no_clust3 differential analysis; (F) Heatmap of clust3 vs no_clust3 differential gene expression.

**Fig.S2:** Clinical phenotypic differences between DAMPs-related subtypes and risk models. (A) Sankey diagram of the relationship between high- and low-risk groups and clinical phenotypes; (B) Differences of risk scores between different phenotypes (*P<0.05; **P<0.01; ***P<0.001; and *** *P < 0.0001).

**Fig.S3:** Correlation analysis between risk scores and 28 immune cell-related scores.
