# Supplementary figures and images for: A damage-associated molecular patterns-related gene signature for the prediction of prognosis and immune microenvironment in children stage III acute lymphoblastic leukemia
Source: Front Pediatr. 2022 Oct 20;10:999684. doi: 10.3389/fped.2022.999684 (PMC9631945; doi:10.3389/fped.2022.999684)

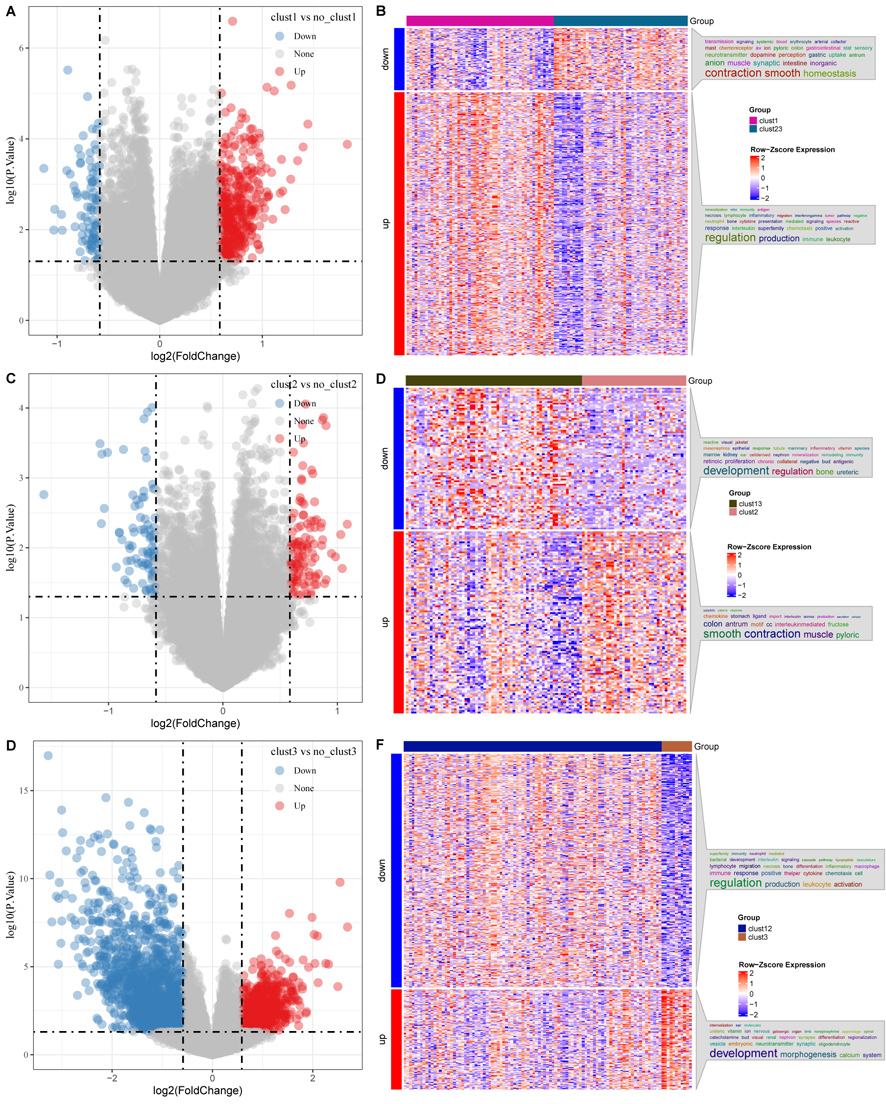

Supplement: Supplementary file 2 [file Image_1.JPEG]

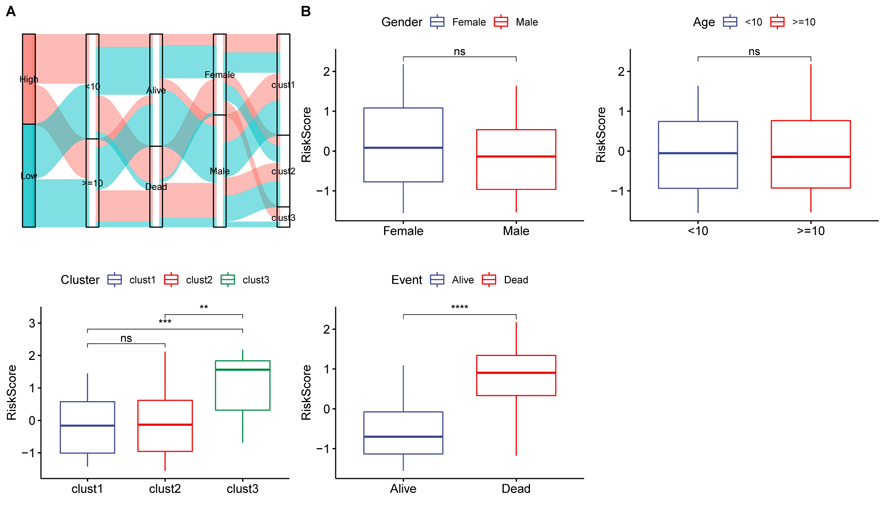

Supplement: Supplementary file 3 [file Image_2.JPEG]

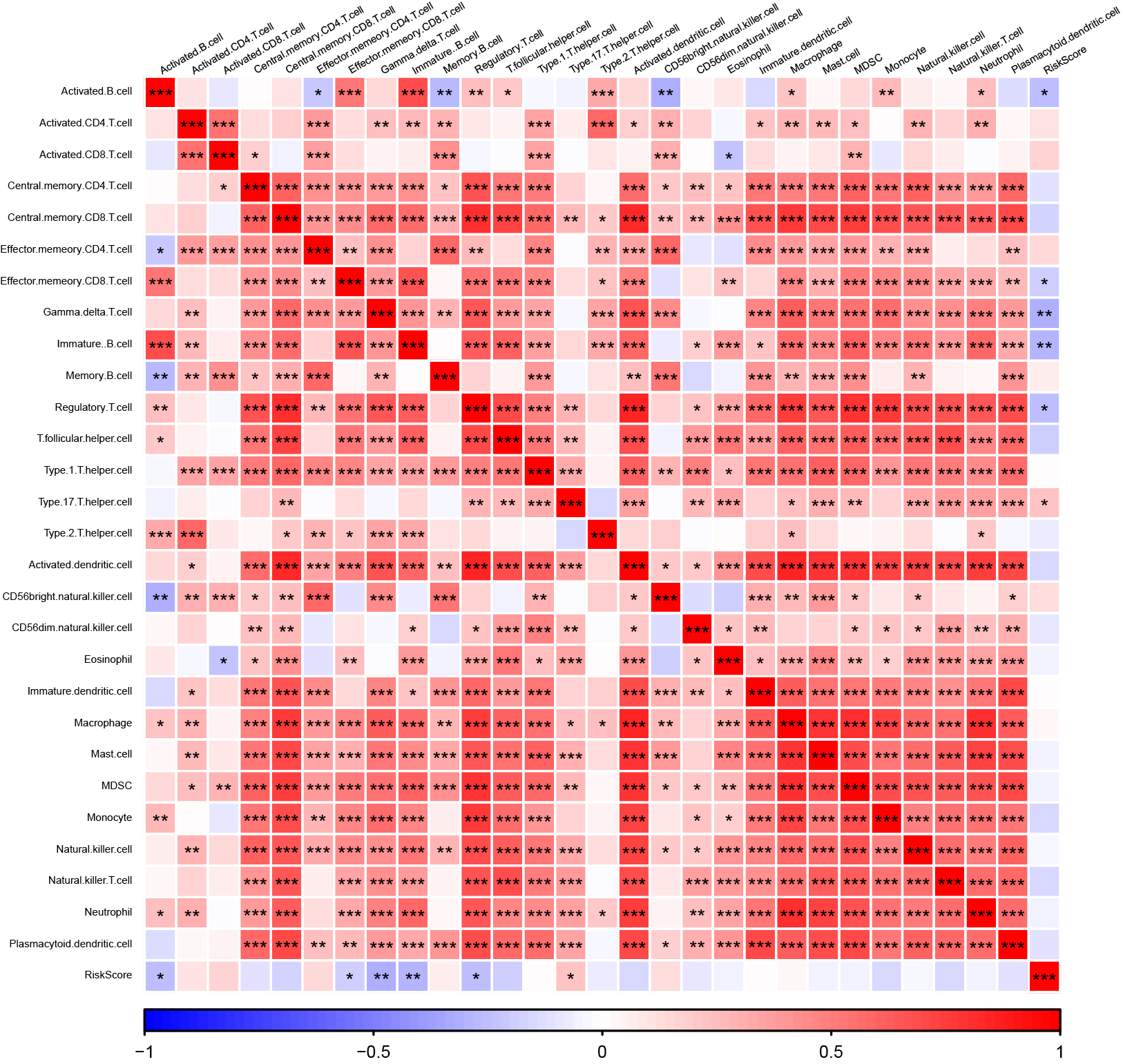

Supplement: Supplementary file 4 [file Image_3.JPEG]
